# Prenatal diagnosis of a de novo tetrasomy 15q24.3‐25.3: Case report and literature review

**DOI:** 10.1002/jcla.23288

**Published:** 2020-03-17

**Authors:** Xiaonan Hu, Leilei Li, Hongguo Zhang, Zhuming Hu, Linlin Li, Meiling Sun, Ruizhi Liu

**Affiliations:** ^1^ Reproductive Medicine & Prenatal Diagnosis Center First Hospital of Jilin University Changchun China; ^2^ Jilin Engineering Research Center for Reproductive Medicine and Genetics Jilin University Changchun China

**Keywords:** 15q duplication, abnormal ultrasound, chromosomal microarray analysis, genetic counseling, prenatal diagnosis

## Abstract

**Background:**

Terminal duplication on chromosome 15q is a rare chromosomal variation. Affected individuals show similar features such as growth dysplasia or the development of frontal bossing, body deformities, facial abnormalities, and genitourinary or cardiovascular disorders. However, it is not yet clear whether such 15q repeats lead to identifiable patterns of clinical abnormalities. Therefore, the purpose of this study was to analyze the prenatal diagnostic results and clinical manifestations of a fetus with 15q duplication and to summarize the literature.

**Methods:**

The case was a fetus at 28 weeks of gestation. The risk of Down syndrome from second‐trimester screening was 1/140. Prenatal ultrasound and amniocentesis were performed, and chromosomal microarray analysis (CMA) was used for genetic analysis.

**Results:**

The fetus had abnormal clinical features, including intracardiac echogenic focus in the left ventricle, an aberrant right subclavian artery, and growth delay. The fetal chromosomal karyotype was 46,XX,15q?,12q?,21pstk+, and CMA revealed a 10.163 Mb duplication at 15q24.3‐q25.3. The couple chose to terminate the pregnancy after careful consideration.

**Conclusions:**

The combination and rational application of cytogenetics technology and molecular genetics technology such as CMA will open up the field of clinical application and provide useful genetic counseling for parents of fetuses carrying such chromosomal duplications.

## 1 INTRODUCTION

Cases of distal 15q chromosomal duplication have been reported in the literature but are very uncommon. After the first case with a duplication of distal 15q was reported in 1974,^1^ approximately 100 cases have been documented, but with few de novo duplications as in the present case. Subsequent case studies showed that chromosome region 15q11‐q13 was a hot spot for chromosomal duplication.^2^, ^3^ However, there are few de novo duplications in the region of 15q24‐qter.^3^ Previous studies have described some cases of 15qter duplication characterized by postnatal or prenatal overgrowth, craniofacial and skeletal malformations, developmental delay, and genital abnormalities. The significant abnormalities in fetal growth and development, and the formation of congenital malformations are caused by the abnormal expression of genes located in the 15qter region such as *LINGO‐1*, *CSPG4*, *MTHFS*, *KIF7*, *CHD2,* and *IGF1R*.^4^, ^5^, ^6^ Moreover, the range and severity of symptoms, and physical findings are closely related to the length and location of the duplicated region of chromosome 15q, and these can vary from case to case.^7^ For example, patients with 15q duplications exhibit some clinical phenotypes that are opposite to overgrowth, such as postnatal or prenatal growth restriction and developmental delay.^8^, ^9^, ^10^, ^11^, ^12^

Here, we reported a fetus with a double de novo duplication of chromosome 15q24.3‐q25.3, producing abnormal sonography findings. The duplication was detected by chromosomal microarray analysis (CMA), and we identified 19 potentially pathogenic genes including *LINGO‐1* and *MTHFS* using the DECIPHER genome browser (https://decipher.sanger.ac.uk). To the best of our knowledge, no case of partial tetrasomy of 15q24.3‐q25.3 has been reported previously.

## 2 MATERIALS AND METHODS

### 2.1 Case report

**Figure 1:**
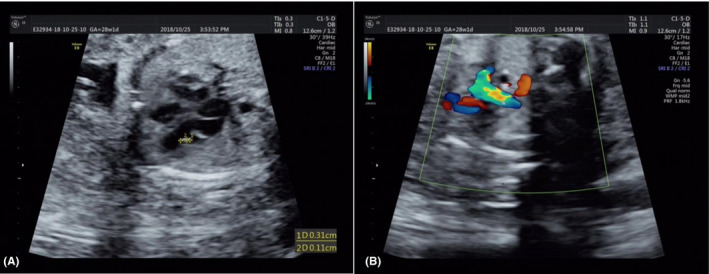
Prenatal ultrasound findings at 28 wk of gestation: A, Intracardiac echogenic focus in the left ventricle; B, Aberrant right subclavian artery

A 28‐year‐old primigravid woman underwent amniocentesis for prenatal diagnosis at 28 weeks of gestation because second‐trimester screening for Down syndrome indicated a high risk (1/140), calculated from abnormal maternal serum screening markers. The multiple of median (MoM) values of maternal serum screening markers were as follows (Table 1): 0.707 MoM for free beta‐human chorionic gonadotropin (Free β‐hCG), 0.503 MoM for unconjugated estriol (uE3), and 0.362 MoM for alpha‐fetoprotein (AFP). Noninvasive prenatal testing was performed at 16^+1^ weeks of gestation (ie, second trimester), but the results showed a low risk of chromosomal aneuploidies. Fetal exfoliated cells in amniotic fluid were used for karyotyping and CMA. Color Doppler echocardiography at 28 weeks of gestation revealed an intracardiac echogenic focus in the left ventricle (Figure 1A) and an aberrant right subclavian artery (Figure 1B). Table 1 shows the results of systemic ultrasonography at 28 weeks of gestation. Fetal abdominal circumference, head circumference, humerus length, and weight were all low for gestational age (<10th centile).^13^, ^14^, ^15^ Hence, the fetus might have experienced intrauterine growth restriction. The fetus's parents were not consanguineous and were healthy. The mother denied being exposed to teratogenic agents or irradiation, or using nicotine, alcohol, or caffeine during the pregnancy. No family history of genetic disease, congenital malformations, or diabetes mellitus was recorded. The study protocol was approved by the Ethics Committee of the First Hospital of Jilin University, and written informed consent was obtained from the couple. Informed consent for publication of this case has also been provided by the couple.

**Table 1:** Maternal serum screening results and ultrasound indicators of fetal size

Indicator	Values	Normal range
Maternal serum screening results		
AFP (MoM)	0.362	0.7‐2.5
Free β‐hCG (MoM)	0.707	0.25‐2.0
uE3 (MoM)	0.503	0.5‐2.0
DS risk	1/140	＜1/270
Evaluation of amniotic fluid		
Maximum deepest vertical pocket (cm)	2.91	2‐8
Amniotic fluid index (AFI) (cm)	8.53	5‐24
Fetal heart rate (FHR) (times per minute)	140	110‐160
Ultrasound indicators of fetal size (28 wk)		(10‐90th centiles)^13^, ^14^, ^15^
Biparietal diameter (cm)	7.02	6.8‐7.5
Femur length (cm)	5.26	5.0‐5.5
Abdominal circumference (cm)	20.78	22.6‐25.2
Head circumference (cm)	25.09	25.2‐27.2
Humerus length (mm)	43.9	45.3‐51.7
Fetal weight (g)	963	995‐1404

Abbreviations: AFP, alpha‐fetoprotein; DS, Down syndrome; Free β‐hCG, free beta‐human chorionic gonadotropin; MoM, multiple of median; uE3, unconjugated estriol.

### 2.2 Cytogenetic examination

According to the standard operating manual of our center's cytogenetics laboratory, at least 20 G‐banded metaphases were used for karyotyping. The karyotype description refers to the International System for Human Cytogenetic Nomenclature (ISCN 2013).^16^ Because of the abnormal karyotype of the fetus, the parents were recalled for further such tests after obtaining written informed consent.

### 2.3 Chromosome microarray and data analysis

Ultrasound‐guided amniocentesis was performed to extract about 10 mL of amniotic fluid for CMA. DNeasy Blood & Tissue kits (Qiagen GmBH) were used to extract genomic DNA according to the manufacturer's instructions. A NanoDrop ND‐2000 spectrophotometer (Thermo Fisher Scientific) was used to quantify the DNA. The potential copy number variations (CNVs) were detected using an Affymetrix CytoScan750K_Array (Affymetrix). DNA processing included digestion, joining, breaking, marking, hybridization, staining, and scanning. Software of the chromosome analysis suite (ChAS) was used to analyze the data. The array data and genotype‐phenotype correlations were analyzed by using the databases of Genomic Variants (http://dgv.tcag.ca/dgv/app/home; GRCh37/hg19), OMIM (https://omim.org), and DECIPHER (see above).^17^

## 3 RESULTS

**Figure 2:**
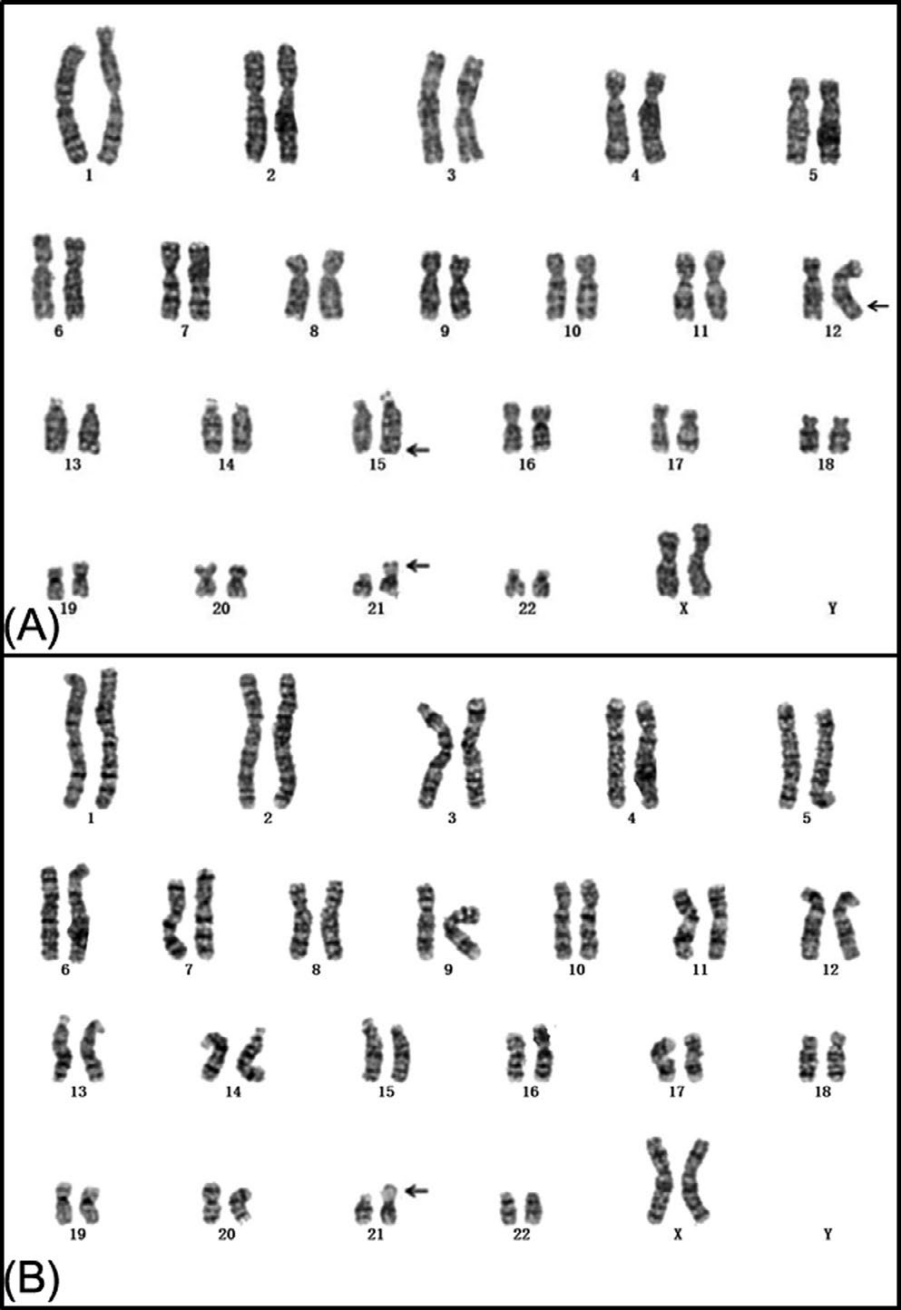
A, Karyotype of the fetus identified by GTG banding technique. B, The mother's karyotype

**Figure 3:**
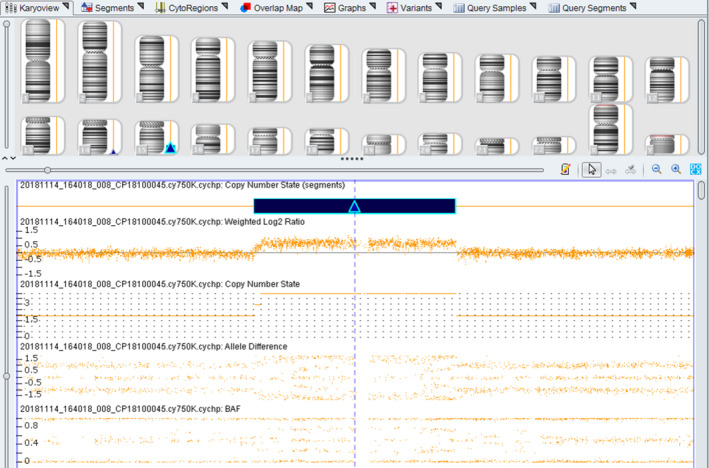
Chromosomal microarray analysis (CMA) on uncultured amniocytes depicted 15q24.3‐q25.3 duplication

Initially, the fetus was diagnosed with an abnormal karyotype of 46,XX,15q?,12q?,21pstk+ (Figure 2A) by routine cytogenetics for prenatal diagnosis. CMA revealed a 10.163 Mb duplication of 15q24.3‐q25.3 at twice (15q24.3‐q25.3 [15:77 456 021‐87618593] × 4) (Figure 3), but no abnormalities were found on chromosome 12q. Conventional cytogenetics demonstrated that the mother had a chromosomal polymorphism of 21pstk+ (Figure 2B) but the father had a normal karyotype. Furthermore, the couple asked for a CMA study, and all results were normal. Thus, the chromosome 15q24.3‐q25.3 duplication of the fetus detected by CMA was a de novo chromosomal variation. Finally, the couple chose to terminate the pregnancy after careful consideration because of the abnormal ultrasonography findings and CMA results.

## 4 DISCUSSION

Trisomy or tetrasomy for chromosome 15qter is very rare; about 30 patients have been described in the literature to date. Affected individuals exhibit similar clinical features, including cephalic or facial deformities, osteoarticular abnormalities, a subarachnoid space, and renal, urogenital or cardiovascular diseases.^18^ Here, we report another fetus with a nonmosaic tetrasomy at 15q24.3‐q25.3 resulting from duplication of chromosome 15qter. The fetus exhibited abnormalities including an intracardiac echogenic focus in the left ventricle, an aberrant right subclavian artery, growth delay and low amniotic fluid index, and a double de novo duplication of 15q24.3‐q25.3 identified by CMA. To illustrate genotype‐phenotype correlations, the similar clinical features of individuals with 15q24‐qter duplications are summarized in Table 2.^4^, ^6^, ^7^, ^10^, ^19^, ^20^, ^21^, ^22^, ^23^, ^24^, ^25^, ^26^ A total of 16 cases listed in Table 2 were pure 15q duplication, and which covers the 15q24.3‐25.3 region. Among the 16 cases, there were 4 cases with tetrasomy for 15qter and 12 cases with trisomy. The detection methods are as follows: 2 cases detected by karyotype, 9 cases by karyotype combined with FISH, and 5 cases detected by CMA.

**Table 2:** Summary of clinical manifestations in patients with terminal duplication on chromosome 15q

References	Yip et al^19^	Blennow et al^20^		Abe et al^21^	Roggenbuck et al^9^			Genesio et al^10^	Schluth et al^22^
Age/sex	NR	caseA 2 y/M	caseB 3 y/F	2 mo/F	case1 died/F	case2 4.5 y/F	case3 21 mo/M	3 wk/F	23 y/M
Duplicated region	15q21‐qter	15q23‐qter	15q24‐qter	15q24.3‐q26.3	15q24‐q26.3	15q24‐q26.3	15q24‐q26.3	15q21.3‐q26.3	15q24.3‐qter
Facial dysmorphism	+	+	+	+	NR	+	+	+	+
Malformation of finger or toe	+	+	+	+	NR	+	+	+	+
Muscular hypotonia	+	NR	NR	NR	NR	NR	NR	+	NR
Convulsive seizures or tremor	+	NR	NR	NR	NR	NR	NR	+	NR
Osteoarticular abnormality	−	+	+	+	NR	−	−	−	+
Arachnodactyly	NR	+	+	NR	NR	−	−	NR	+
Achromatopsia	NR	+	−	NR	NR	NR	NR	NR	NR
Sensorineural hearing defect	+	+	+	NR	NR	+	−	NR	−
Mental retardation	+	+	+	NR	NR	+	NR	NR	+
Postnatal overgrowth	−	+	+	NR	NR	NR	NR	−	−
Developmental or growth delay	+	NR	NR	+	NR	+	−	+	NR
Cardiac Malformations	−	−	−	+	NR	+	+	+	NR
Nural tube defects	−	−	−	−	+	−	−	−	−
Short neck	−	−	−	−	NR	+	−	−	−
Genital abnormality	NR	−	−	NR	NR	+	+	−	NR
Brachycephaly	−	−	−	NR	NR	−	+	+	−
Renal dysplasia	NR	NR	NR	NR	NR	NR	NR	+	+
Intrauterine overgrowth	NR	NR	NR	NR	NR	NR	NR	−	NR
Single umbilical artery	NR	NR	NR	NR	NR	NR	NR	NR	NR

References	Liehr et al^23^	Gutierrez‐Franco Mde et al^24^	Kim et al^7^	O'Connor et al^25^	Chen et al^6^	Ochando et al^4^	Alakbarzade et al^26^	Present case
Age/sex	11 y/F	13 y/F	Newborn/F	Newborn/F	Fetus/F	Fetus/M	Pedigree	Fetus/F
Duplicated region	15q24.1‐qter	15q24‐q26.3	15q24‐q26.3	15q24.2‐q26.3	15q24.2‐q26.2	15q24.3q26.1	15q24.3‐q25.1	15q24.3‐q25.3
Facial dysmorphism	+	+	+	+	NR	NR	−	
Malformation of finger or toe	+	+	+	+	NR	−	NR	
Muscular hypotonia	+	−	NR	NR	NR	NR	+	
Convulsive seizures or tremor	+	−	NR	NR	NR	NR	+	
Osteoarticular abnormality	+	+	+	+	NR	+	NR	−
Arachnodactyly	NR	NR	+	NR	NR	NR	NR	
Achromatopsia	−	−	NR	NR	NR	NR	NR	
Sensorineural hearing defect	−	−	NR	NR	NR	NR	NR	
Mental retardation	+	+	NR	NR	NR	NR	+	
Postnatal overgrowth	NR	−	NR	NR	NR	NR	−	
Developmental or growth delay	+	−	+	NR	NR	+	−	+
Cardiac Malformations	NR	−	+	+	NR	NR	NR	+
Nural tube defects	−	−	−	−	+	−	−	−
Short neck	−	−	−	−	NR	NR	NR	
Genital abnormality	NR	−	NR	+	NR	NR	−	
Brachycephaly	−	−	−	−	NR	NR	−	
Renal dysplasia	NR	−	+	NR	NR	NR	NR	
Intrauterine overgrowth	NR	−	+	NR	NR	−	NR	−
Single umbilical artery	NR	NR	NR	NR	NR	+	NR	−

Abbreviations: −, feature absent; +, feature present; F, female; M, male; NR, not reported.

According to the literature,^27^ we inferred that the clinical features of 15q24‐qter duplication are genetic disorders that may be caused by nonallelic homologous recombination between low‐copy repeats in the region of chromosome 15q24‐qter. The 15q24‐q26 region is one of several hotspots reported, with a high density of chromosome‐specific duplications. Rearrangements of this region have been implicated as a susceptibility factor for panic and phobic disorders with joint laxity.^28^ However, according to case reports, it is not yet clear whether the 15q repeats lead to identifiable patterns of clinical abnormalities.^4^ Zollino et al^5^ summarized 32 patients with 15q duplications and divided them into two groups: The first had duplication at 15q21‐24qter, revealing normal prenatal growth and microcephaly; the second had duplication of 15q25‐26qter, leading to macrocephaly, prenatal overgrowth, and craniosynostosis. Gutierrez‐Franco Mde et al^24^ reported a case of trisomy of distal 15q with overgrowth and mental retardation. Another case report described a newborn infant with a de novo 15q24‐q26.3 duplication and intrauterine overgrowth.^7^ However, ultrasonography indicated that the fetus in this case might have had growth delay, but there was no sign of head size deformity.

Mental retardation seems to be a common feature of patients with duplication of 15qter,^8^ because several genes related to brain development and function are involved in the 15q24.3‐qter region.^29^ Schluth et al,^22^ Liehr et al,^23^ and Alakbarzade et al^26^ reported cases of clinical manifestations of mental retardation. Another common feature of individuals with duplication of 15qter is cardiac malformation, which has been found in about 50% of the cases.^8^ The types of cardiac malformations include Wolf‐Parkinson‐White syndrome, mitral valve stenosis, Ebstein's anomaly, mitral valve arcade, defects of the atrial and ventricular septum, an atrioventricular canal, subaortic stenosis, patent foramen ovale or ductus arteriosus, cardiomegaly, and an aberrant right subclavian artery (as in the present case).^7^, ^9^, ^10^, ^21^, ^25^, ^30^ Here, we also found cardiac malformations manifested as an intracardiac echogenic focus in the left ventricle. Obviously, we could not evaluate whether the fetus had mental retardation, because this can only be observed after birth and ultrasonography can only indicate a structural abnormality.

**Figure 4:**
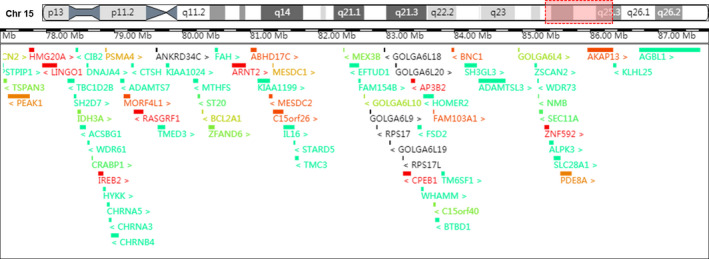
The involving genes contained in the region of 15q24.3‐q25.3 (15:77456021‐87618593). The figure is modified from the DECIPHER genome browser

ADAMTSL3 (cytogenetic location: 15q25.2; OMIM #609199), a gene associated with the duplication of chromosome 15qter has been identified as a potential cause of cardiac and vessel malformations.^18^, ^31^, ^32^ Overexpression of the ADAMTSL3 gene has also been thought to interfere with kidney function.^32^ Genesio et al^10^ reported a female infant with a 15q21.3‐q26.3 duplication and a horseshoe kidney, and Kim et al^7^ reported a newborn with a 15q24‐q26.3 duplication with hydronephrosis. However, no fetal kidney abnormality was found in our case. All the genes involved in this region are shown in Figure 4. The 15q24.3‐q25.3 region contains 19 morbid genes involved in morbidity, namely *LINGO1*,* CIB2*,* IREB2*,* CHRNA5*,* CHRNA3*,* MIR184*,* MTHFS*,* FAH*,* ARNT2*,* MESDC2*,* EFTUD1*,* RPS17*,* RPS17L1*,* AP3B2*,* HOMER2*,* WDR73*,* ALPK3*,* SLC28A1*, and *AGBL1.* The corresponding phenotype of these genes and explanations are summarized in Table 3. *LINGO‐1* and *MTHFS* have been shown to be related to the clinical manifestations.

**Table 3:** Morbid genes in the region of 15q24.3‐25.3 and the associated phenotype

Gene	location	OMIM	Explanation	Phenotype
*LINGO1*	15q24.3	609791	Leucine‐Rich Repeat‐ And Ig Domain‐Containing Nogo Receptor‐Interacting Protein 1	Mental retardation, autosomal recessive 64
*CIB2*	15q25.1	605564	Calcium‐ And Integrin‐Binding Protein 2	Deafness, autosomal recessive 48, Usher syndrome, type IJ
*IREB2*	15q25.1	147582	Iron responsive element binding protein 2	Neurodegeneration, early‐onset, with choreoathetoid movements and microcytic anemia
*CHRNA5*	15q25.1	118505	Cholinergic Receptor, Neuronal Nicotinic, Alpha Polypeptide 5	Smoking as A Quantitative Trait Locus 3; SQTL3
*CHRNA3*	15q25.1	118503	Cholinergic Receptor, Neuronal Nicotinic, Alpha Polypeptide 3	Smoking as A Quantitative Trait Locus 3; SQTL3
*MIR184*	15q25.1	613146	Micro Rna 184	EDICT syndrome
*MTHFS*	15q25.1	604197	5,10‐Methenyltetrahydrofolate Synthetase	Neurodevelopmental disorder with microcephaly, epilepsy, and hypomyelination
*FAH*	15q25.1	613871	Fumarylacetoacetate Hydrolase	Tyrosinemia, type I
*ARNT2*	15q25.1	606036	Aryl Hydrocarbon Receptor Nuclear Translocator 2	Webb‐Dattani syndrome
*MESDC2*	15q25.1	607783	Mesoderm Development Candidate Gene 2	Osteogenesis imperfecta, type XX
*EFTUD1*	15q25.2	617538	Elongation Factor‐Like Gtpase 1	Shwachman‐Diamond syndrome 2
*RPS17*	15q25.2	180472	Ribosomal Protein S17	Diamond‐Blackfan anemia 4
*RPS17L1*	15q25.2	180472	Ribosomal protein S17a‐Like 1	Diamond‐Blackfan anemia 4
*AP3B2*	15q25.2	602166	Adaptor‐Related Protein Complex 3, Beta‐2 Subunit	Epileptic encephalopathy, early infantile, 48
*HOMER2*	15q25.2	604799	Homer, Drosophila, Homolog Of, 2	Deafness, autosomal dominant 68
*WDR73*	15q25.2	616144	Wd Repeat‐Containing Protein 73	Galloway‐Mowat syndrome 1
*ALPK3*	15q25.3	617608	Alpha Kinase 3	Cardiomyopathy, familial hypertrophic 27
*SLC28A1*	15q25.3	606207	Solute carrier family 28, member 1	Uridine‐cytidineuria
*AGBL1*	15q25.3	615496	Atp/Gtp‐Binding Protein‐Like 1	Corneal dystrophy, Fuchs endothelial, 8

Traditional chromosome banding and karyotyping have always been the gold standard of cytogenetics and have irreplaceable advantages.^33^ It is generally known that chromosome rearrangements of <10 Mb are hard to recognize using routine karyotyping. CMA can be used to describe the location, functional genes involved, and size of the rearranged region more precisely.^34^ However, it has some limitations.^33^ In the present case, the fetal karyotype was 46,XX,15q?,12q?,21pstk+. The mother was 46,XX,21pstk+, and the father was normal (46,XY). The fetal 21pstk+ chromosomal polymorphism was inherited from the mother, but this is considered to have no detrimental phenotypic effect.^35^ Furthermore, CMA results demonstrated a de novo duplication of 15q24.3‐25.3. Hence, further prenatal counseling for the pregnant woman and her family could be given appropriately. However, the results were complicated; CMA works by detecting imbalances in DNA copy numbers, or CNVs.^36^ Therefore, based on our results and theoretical knowledge, we speculate that the 15q duplication fragment might have been a partially balanced translocation with 12q, which is probably why CMA could not detect a chromosome 12q abnormality. Clearly, the origin of any abnormal fetal chromosome 12q found by karyotyping needs to be verified by other techniques, such as fluorescence in situ hybridization (FISH). Further FISH verification can indeed identify the location of the duplication and verify the diagnostic results. However, there were no remaining samples after completing the karyotype and CMA, and the fetus had induced labor. This is the limitation of the present study.

The clinical manifestations presented here were linked to 15q a duplication in the fetus detected by CMA. As a molecular genetics detection technique, CMA can detect many chromosome structural abnormalities, but the technology has limitations. The combination and rational application of cytogenetics and molecular genetics technologies will undoubtedly open up the field of clinical application for such anomalies.

## CONFLICT OF INTEREST

The authors declare that there is no conflict of interest.

## AUTHORS CONTRIBUTIONS

Xiaonan Hu and Zhuming Hu involved in writing‐original draft. Meiling Sun, Hongguo Zhang, and Linlin Li involved in investigation. Leilei Li and Zhuming Hu involved in methodology. Ruizhi Liu involved in funding acquisition. Ruizhi Liu and Leilei Li. involved in writing‐review and editing.
